# Spatial Frequency-Based Analysis of Mean Red Blood Cell Speed in Single Microvessels: Investigation of Microvascular Perfusion in Rat Cerebral Cortex

**DOI:** 10.1371/journal.pone.0024056

**Published:** 2011-08-24

**Authors:** Joonas Autio, Hiroshi Kawaguchi, Shigeyoshi Saito, Ichio Aoki, Takayuki Obata, Kazuto Masamoto, Iwao Kanno

**Affiliations:** 1 Department of Biophysics, Molecular Imaging Center, National Institute of Radiological Sciences, Anagawa, Chiba, Japan; 2 Center for Frontier Science and Engineering, University of Electro-Communications, Chofu, Tokyo, Japan; University of Arizona, United States of America

## Abstract

**Background:**

Our previous study has shown that prenatal exposure to X-ray irradiation causes cerebral hypo-perfusion during the postnatal development of central nervous system (CNS). However, the source of the hypo-perfusion and its impact on the CNS development remains unclear. The present study developed an automatic analysis method to determine the mean red blood cell (RBC) speed through single microvessels imaged with two-photon microscopy in the cerebral cortex of rats prenatally exposed to X-ray irradiation (1.5 Gy).

**Methodology/Principal Findings:**

We obtained a mean RBC speed (0.9±0.6 mm/sec) that ranged from 0.2 to 4.4 mm/sec from 121 vessels in the radiation-exposed rats, which was about 40% lower than that of normal rats that were not exposed. These results were then compared with the conventional method for monitoring microvascular perfusion using the arteriovenous transit time (AVTT) determined by tracking fluorescent markers. A significant increase in the AVTT was observed in the exposed rats (1.9±0.6 sec) as compared to the age-matched non-exposed rats (1.2±0.3 sec). The results indicate that parenchyma capillary blood velocity in the exposed rats was approximately 37% lower than in non-exposed rats.

**Conclusions/Significance:**

The algorithm presented is simple and robust relative to monitoring individual RBC speeds, which is superior in terms of noise tolerance and computation time. The demonstrative results show that the method developed in this study for determining the mean RBC speed in the spatial frequency domain was consistent with the conventional transit time method.

## Introduction

Prenatal exposure to X-ray irradiation is a leading cause of postnatal development deficits such as a decrease in brain size and retardation of behavioral and mental activity [1]. Recently, we found that X-ray irradiation exposure (1.5 Gy) on the 15th day of pregnancy impaired the development of vascular endothelial cells and cerebral arteriogenesis in postnatal rat brains [2]. Further, following prenatal exposure to irradiation, newborn rats showed reduced cerebral blood flow (CBF), which was only half of that observed in age-matched non-exposed rats [2]. These findings suggest that immature development of the brain vasculature, including cerebral hypo-perfusion, is of etiological importance in the delayed development of the central nervous system (CNS) observed in subjects following prenatal exposure to X-ray irradiation.

Because the CBF reflects a product of cortical blood volume per unit tissue volume and cross-sectional blood velocity in the vessels, the cerebral hypo-perfusion observed in the exposed subjects could be due to low blood volume (i.e., decrease in capillary density) in the parenchyma tissue relative to non-exposed subjects, or it could be due to a decrease in blood velocity through the parenchyma capillaries. In the former case, low blood volume may result from an immature development of the microvasculature. In the latter case, a decline in blood velocity can be interpreted as a higher resistance in the cerebral circulation in the exposed subjects, which can be attributed to a narrow lumen space of cerebral arteries or low demand of metabolic activity in parenchyma tissue. In either case, it is necessary to measure the blood velocity in the parenchyma capillaries directly to further determine the source of the hypo-perfusion observed in the prenatal exposed subjects.

Using a variety of fast-scanning optical microscopic techniques, previous studies have shown that the mean red blood cell (RBC) speed in cortical microvessels ranged from 0.4 to 1.5 mm/sec in anesthetized rats [3]-[7]. However, these studies were limited to microvessels located on the cortical surface, but not in parenchyma capillaries, where neural processes and metabolic activity occur. Alternatively, Kleinfeld et al. [8] first used two-photon microscopy to directly visualize RBC movement through the parenchyma capillaries up to a depth of 600 µm. Two-photon microscopy has an increase in the depth of tissue penetration into biological tissue as compared to other most commonly used optical microscopes [9], [10]. The near-infrared excitation wavelengths used in two-photon microscopy allows for high penetration of the light into the tissue due to low absorption and scattering effects [11]. However, the depth penetration of two-photon imaging is decreased in circumstances of increased tissue absorption and scattering [12], [13], which makes analyzing small targets, i.e., moving RBCs, difficult, such as in disease model animals where the scattering and/or absorption of light by the tissue is high.

To overcome the potential limitations in analyzing RBC speed from poor contrast-to-noise ratio images in disease model animals, we developed an analytical method based on a two-dimensional fast Fourier transform (FFT) approach. This method is similar to that used in a recent study by Drew et al. [14] who used the Radon transform to characterize the spatial pattern of the RBC traces. In their study, it was shown that a coordinate-transform method is superior in terms of noise tolerance for the extraction of RBC slope from line-scanned vascular images, as compared to the original approach with a singular value decomposition (SVD) method [8]. The 2D FFT approach presented here directly utilized raw line-scanned images, which contained vascular and non-vascular signals. The method was then applied to determine the mean RBC speed in the cerebral cortex in rats receiving prenatal X-ray irradiation (1.5 Gy) according to our previous study [2]. These results were further compared with the conventional method for determining the microvascular perfusion based on the arteriovenous transit time (AVTT) [15]-[17] in both X-ray exposed and non-exposed rats. Using this new analytical approach to determine the microvascular RBC speed and conventional AVTT measurements, we consistently found approximately 40% lower microvascular perfusion in the cerebral cortex of prenatally exposed rats as compared to non-exposed rats.

## Results

### RBC speed in single microvessels

**Figure 1:**
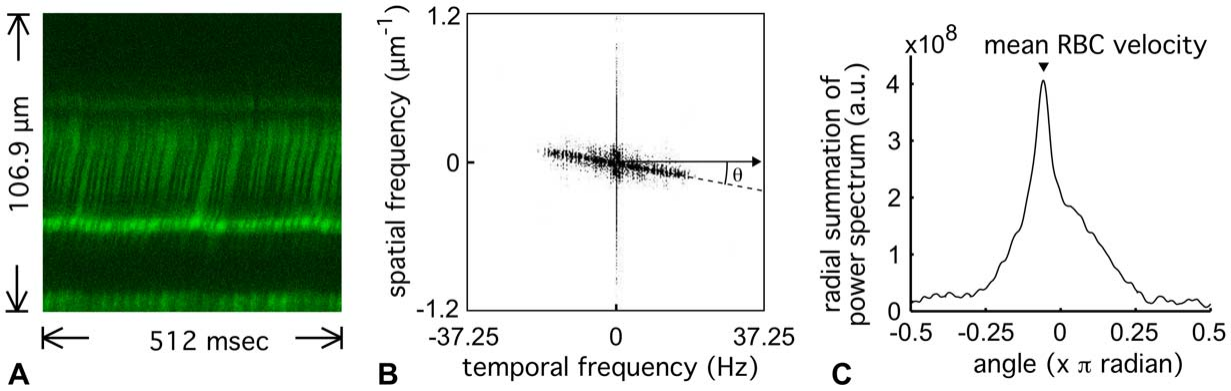
Representative image and measurement of RBC speed in single microvessels of the exposed animals. (A) A raw image of RBC moving through a microvessel was obtained using a line-scan mode with two-photon microscopy. The representative image showed 512 lines captured at the center of the target microvessel in parallel to the longitudinal direction. The x-axis represents Δt of 1 msec/pixel (time domain), and the y-axis is Δx of 0.20 µm/pixel (spatial domain). The green color represents the measured intensity of the fluorescent signal that originated from the injected plasma marker. Dark streaks observed around the center of the image were mainly caused by unlabelled RBC motions, and their slopes reflect a speed of the RBC motions that were parallel to the vessel length (a scan direction). (B) Power spectrum image. The image represents a FFT image constructed from the original 512 by 512 pixel image (A). The power spectrum was used to characterize a periodic pattern of the pixel intensity distribution represented in the raw image (A). A slanted line reflects a direction preference, perpendicular to the RBC traces (i.e., a slope of dark streaks), which appeared in the original image. The angle between this slanted line and the temporal axis was used to calculate the mean RBC speed (see text). (C) The spatial pattern of the pixel intensity distribution converted by the FFT analysis. Summation of the power spectrum at each angle was calculated for all directions (-0.5 to 0.5 π), and the angle that had the maximum power (arrow head) was used to calculate mean RBC speed (1.1 mm/sec in this representative image) (see Eq. 1).

**Figure 2:**
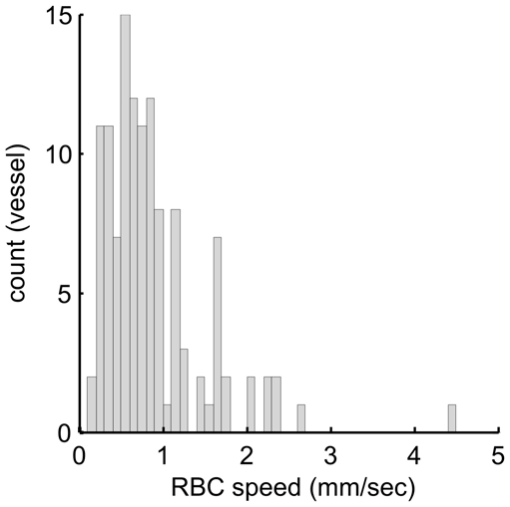
Mean RBC speed in single microvessels. The histogram shows the frequency distribution of the mean RBC speed obtained from all 121 vessels in the exposed rats (N = 4). The mean RBC speed ranged from 0.2 to 4.4 mm/sec (minimum to maximum), and the mean of all measurements was 0.9±0.6 mm/sec.

Figure 1A shows a representative raw image of an RBC trace using the two-photon line-scanning method at a single location in the parenchyma microvessel. Dark streaks in the image represent the unlabelled RBC moving through the vessel in a longitudinal direction. Figure 1B shows a power spectrum image calculated from the 512 by 512 pixel image in Fig. 1A. The oblique shape in the FFT image is elongated in the direction perpendicular to the dark streaks in the original image. Changing to polar coordinates in the frequency space, the power spectrum was summed along lines of θ (Fig. 1C, and see Eq. 2). The angle corresponding to the peak of the radial spectrum was then used to calculate the mean RBC speed with Eq.1. The mean RBC speed in this representative image was calculated to be 1.1 mm/sec. Under our experimental protocol, the time required to compute the mean RBC speed was 0.60 sec per image with the proposed method, which was faster than a method employing SVD (4.74 sec per image), and almost equivalent to a method using the Radon transform (0.75 sec per image). The population data (121 vessels from four exposed rats) showed that the obtained mean RBC speed ranged from 0.2 to 4.4 mm/sec, and the mean of all measurements was 0.9±0.6 mm/sec (Fig. 2).

### Cortical AVTT measurements

**Figure 3:**
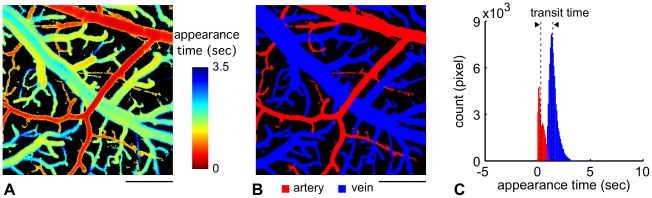
Representative image and measurement of AVTT. (A) A representative image of the appearance time obtained from one animal. Time-lapse imaging was performed at the cortical surface with a frame-capture rate of 14.2 frame/sec. The field of view was 512 by 512 pixels, and in-plane resolution was 3.6 µm/pixel. The pixel-basis analysis of appearance time was performed (see text). The image showed a continuity of appearance time in each vascular segment. An early appearance time (red) mostly represented an arterial flow, whereas a late appearance time (yellow to blue) resulted from venous flow. The color bar indicates the image acquisition time. Scale bar: 0.5 mm. (B) Segmentation of arterial and venous compartments. The arterial (red) and venous (blue) blood vessel areas were determined based on the spatial continuity of the appearance time observed along a longitudinal direction of the vessels (see text). Scale bar: 0.5 mm. (C) AVTT. The histogram represents the frequency distribution of appearance time observed in respective arterial (red) and venous (blue) areas. A total of 37,638 and 97,272 pixels were counted for arterial and venous segments, respectively, in this representative animal. Mean appearance time was observed as 0.29±0.28 and 1.43±0.48 sec in the arterial and venous segments, respectively, and thus, AVTT was 1.14 sec (a width between dashed vertical lines).

Figure 3A shows an image of the appearance time measured on the cortical surface of a representative non-exposed animal. Based on the spatial continuity of the appearance over time, arterial and venous segments were determined (37,638 and 97,272 pixels in this representative image, which accounted for 14% and 37% of total pixel numbers, respectively, Fig. 3B). In this rat, the mean appearance time was 0.29±0.28 and 1.43±0.48 sec in the arterial and venous segments, respectively (see Fig. 3C). Consequently, the AVTT was calculated as 1.14 sec. In addition, the median of the appearance time (0.21 and 1.33 sec in arterial and venous segments, respectively) was used to compare transit times. The AVTT was 1.12 sec, which was similar to the results obtained from the mean value. Therefore, the AVTT was calculated using the mean values.

**Figure 4:**
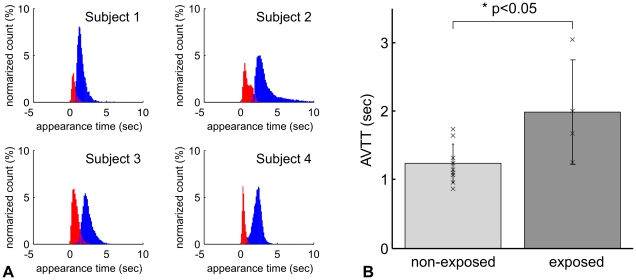
A comparison of mean AVTT. (A) The normalized histogram of the appearance time in all exposed rats (N = 4). The consistent distribution pattern of the appearance time was observed for both the arterial (red) and venous (blue) segments. (B) Population data on mean AVTT. Mean of AVTT was 1.2±0.3 and 1.9±0.6 sec in non-exposed (N = 9) and radiation exposed animals (N = 4), respectively. There were statistically significant differences between the two groups (p<0.05).

A clear separation in the appearance time between the arterial and venous segments was consistently observed in all four exposed animals (Fig. 4A). The mean area of the observed vascular segments was 15,789±10,909 and 34,355±11,539 pixels in the arterial and venous segments, respectively, corresponding to 6±4% and 13±4% of the total number of pixels (262,144) in the image, respectively. In contrast, the results for the non-exposed animals were 12±4% and 30±9% of the pixels occupied by the arterial and venous segments, respectively. Interestingly, the mean ratio of the areas covered by the arterial and venous segments was similar for the exposed (1∶2.2) and non-exposed (1∶2.4) animals.

**Figure 5:**
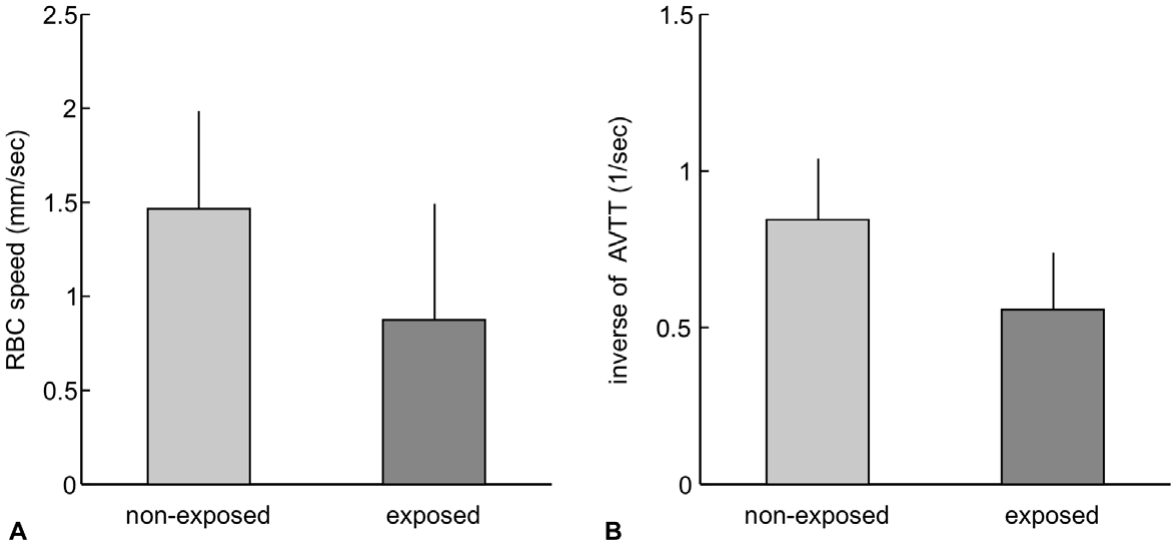
A comparison of capillary blood speeds. (A) Mean capillary RBC speed. The mean capillary RBC speed measured in the present study for the exposed animals (N = 4) was 40% lower than that for the non-exposed animals measured in a previous study (modified from [30]). (B) Inverse of the mean AVTT. The inverse of the mean AVTT of the exposed animals was 34% lower than that of the non-exposed animals. The ratio of exposed to non-exposed animal results was similar for both data sets.

The mean of the AVTT was 1.9±0.6 sec in exposed animals, which was significantly longer than that of non-exposed animals (1.2±0.3 sec, Fig. 4B). To further compare the baseline capillary blood velocities in the exposed and non-exposed animals, the inverse of the mean AVTT was plotted in Figure 5B. Assuming that the tissue blood volume (i.e., a total vessel length) was the same for the two groups, the observed inverse transit times may reflect the mean of capillary blood speed through the parenchyma tissue. Comparison of the inverse transit times for the non-exposed and exposed groups found a ratio of 66%. A comparison for the mean capillary RBC speed measured in the present (exposed) and previous (non-exposed) studies found a similar ratio of 60% (Fig. 5A).

## Discussion

### Technical issues of frequency-based image analysis

Using the spatiotemporal frequency-based approach, the automated image analysis method was developed to measure the mean RBC speed from the population of RBCs moving in single microvessels of the cerebral cortex (Figs. 1, 2). The advantages of the present approach are the following: i) the mean RBC speed can be determined from images that contain a population of RBCs without distinguishing individual cells, such as for low signal-to-noise images, ii) the computation time required for the calculation is significantly improved relative to the previously-reported method with SVD, and iii) the algorithm is simple and no prior information is required, which makes it easy to implement. Based on a space-time image obtained by a line-scanning of RBC motions along the vessel, early studies have established a method that enabled RBC speed to be determined from the slope of the RBC displacement per certain time intervals (i.e., scanning intervals) [4], [17]. Later studies have presented a variety of image-based approaches, using SVD [8], [18], two-dimensional image autocorrelation [19], Hough transform [20], and Radon transform [14], [21], to extract the average slope. In particular, the SVD and Radon transform methods have been widely used to automatically determine dynamic changes in brain capillary RBC speeds [22]-[24]. For dynamic imaging, computation time is a key issue for achieving real time monitoring. We compared the computation time required for the method proposed in this paper and these alternative methods, and found that under our experimental conditions the proposed method is about 8 times faster than the SVD and equivalent to or slightly faster (1.25 times) than the Radon transform methods. With a Radon transform approach and simulated data degraded with various levels of noise, Drew et al. [14] showed that the coordinate-transform method has some advantages over the SVD. The 2D FFT presented here utilized the periodic pattern of the pixel intensity distribution (i.e., RBC streaks), and thus is relatively insensitive to signal arising from the non-vascular areas. This means that there is no need to crop the vascular areas from the raw image to detect RBC streaks. This is demonstrated in our supplementary results where it is shown that when the image covered relatively large non-vascular areas, the present method found a single unique peak, whereas the Radon transform method did not (Fig. S1). An algorithm that reliably provides a unique peak is important for running the automated analysis, and no requirement to crop the images would be beneficial in reducing user bias and the number of steps in image processing. Further, specificity of the present method to the RBC streaks in the image could be used to reduce non-vascular signals by applying a bandpass filter in the frequency domain. This approach would be also useful as a pre-processing step (denoising) prior to identifying individual RBCs for counting [25], fitting [26], and tracking [27]-[29].

In contrast, one limitation of the frequency-based approach is that the method is only applicable to an image that contains a single pattern of RBC traces. If the images contain multiple patterns of RBC traces, such as due to temporal fluctuations and spatial variations of RBC movements through a single microvessel, it is necessary to assign an appropriate region of interest and time window (see also Fig. S2). The spatiotemporal dynamics of the RBC behaviors, such as during spontaneous fluctuations and response to neural functions in both prenatal exposed and non-exposed subjects, must be investigated in future studies.

### Radiation effects on cerebral microcirculation

We observed that the mean RBC speed was 0.9±0.6 mm/sec in the exposed rats (Fig. 2), which was approximately 40% lower than that in isoflurane-anesthetized normal rats (Fig. S3 modified from [30]). Other studies have shown that the mean RBC speed in the microvessel on the cortical surface and parenchyma tissue ranged from 0.4 to 1.5 mm/sec as measured in anesthetized rats [3]-[8], [22], [31]. This large variation could be due to animal physiology from various labs and/or methods used to determine the RBC speed. In our previous study, we found that anesthesia significantly impacted the mean RBC speed in cortical microvessels; 0.4±0.4 mm/s and 1.5±0.4 mm/s under alpha-chloralose and isoflurane, respectively [30]. These findings are consistent with other reports that examined the anesthesia-dependence of CBF in rats [32], [33]. The CBF in the somatosensory cortex of rats under isoflurane was shown to be equivalent to the non-anesthetized awake rats (130-150 ml/ 100g/ min) [34], indicating that isoflurane anesthesia is the preferred anesthetic to preserve normal CBF characteristics [35]. Using a high-speed confocal laser scanning microscope, it has been shown that the high-frame rate measurements (500 frame/s) recorded a 2.2 mm/s average for RBC speed (ranged from 0.8 to 6.6 mm/sec) in rats and mice [36], [37]. Therefore, our RBC measurements were further compared with the conventional method for microvascular perfusion measurements [15], [16]. In the present study, a fluorescent dye was injected into the femoral vein to measure the cortical AVTT. During its passage to the measurement site in the cortex the concentration of the dye will be diluted with blood, and this might complicate the separation of artery and venous segments. To avoid this possibility, we used the earliest image where the fluorescent signal appeared in a particular vessel for artery-vein segmentation (Fig. 3). Other studies have injected dye directly into the internal carotid artery [38], [39], which allows the arrival of the dye at the imaging area of the cortex to be approximated by an impulse function (Dirac's delta). From preliminary tests with dye injection into the internal carotid artery, we found the mean AVTT to be 1.3±0.6 sec for the normal rats (N = 3), which was in good agreement with the present results (1.2±0.3 sec) obtained with dye injection into the femoral vein of the non-exposed rats. The intravenous injection is less-invasive than arterial injection methods, and thus has the advantage of permitting repeated long-term measurements of cerebral microvascular perfusion [40]. As a non-invasive technique, laser-Doppler flowmetry (LDF) might be considered as a good alternative to repeated long-term CBF measurements with a dye. However, LDF measures only relative changes with respect to the baseline CBF condition and thus may not be suitable for subject comparisons of stationary CBF between normal and disease states.

For the group comparisons, we assumed that blood volume per unit tissue was equivalent for the exposed and non-exposed animals. However, if the total vessel length is longer in the exposed rats, this might explain the longer mean AVTT that was observed. However, since our MR data showed that the cortical thickness in the exposed animals was only 36% of that of age-matched non-exposed animals (data not shown), this possibility can be ruled out. Measurements of the RBC speed and inverse of mean AVTT consistently showed an approximately 40% decline in cortical microvascular perfusion in the exposed rats in comparison to the non-exposed rats (Figs. 4, 5). These findings are also in good agreement with our previous measurements of CBF using magnetic resonance imaging (MRI) that showed that exposed newborn rats (postnatal 2 weeks) had approximately 50% of the CBF of the age-matched non-exposed rats [2].

The cerebral hypo-perfusion observed in the prenatal exposed rats could be due to the immature development of vascular systems and/or secondary effects of a lower energy demand in parenchyma tissue. In a previous study, with MR angiography, we found that the diameter of the middle cerebral artery was 63% smaller in the prenatally-exposed rats (0.24±0.02 mm) in comparison to the non-exposed age-matched animals (0.38±0.02 mm) [2]. In agreement with this finding, the present study showed that the area covered by arterial vessels on the cortical surface was almost half in the exposed animals relative to the non-exposed ones. Since the general physiology (MABP and heart rate) was not significantly different between the two groups, the possibility that the effects of radiation exposure on the CBF are due to general ill-health or lower cardiac output in the animals can be ruled out. Previous and present findings indicate that shrinkage of the major cerebral arteries could increase vessel resistance and result in parenchymal hypo-perfusion. Further technical improvement of cerebro-microvascular angiography would help highlight the main source of increased resistance at a microscopic scale.

In addition, no leakage of the fluorescent marker into the extravascular space was observed (data not shown), which indicates an intact blood-brain barrier (BBB) for the exposed as well as non-exposed animals. In a previous study, albumin-staining histology was performed to check the permeability of the BBB for prenatally-exposed rats [2]. The results showed that the number of albumin-positive cells in the cortex drastically decreased 2 weeks after birth for both exposed and non-exposed animals [2]. The findings also indicate that the function of the vascular endothelium (i.e., BBB permeability) developed normally for the prenatally exposed animal brains. In contrast, other studies have shown that X-ray exposure (60 Gy) of adult rats provoked disruption of BBB functions [41]. The discrepancy between the present and previous studies could be due to different dosage (1.5 Gy vs. 60 Gy) and/or age of exposure (prenatal vs. adult). In our studies, we also found that the mean density of laminin-positive cells (i.e., vascular endothelium cells) was significantly lower for the exposed (3,300 cells/mm^2^) relative to the non-exposed (5,200 cells/mm^2^) 2-week-old rat cortex [2]. The findings are consistent with results for adult rats and mice showing that loss of endothelial cells depends on X-ray irradiation dose and time after exposure [42], [43]. Because the vascular endothelial cells belong to a category of high radiosensitivity cells [44], these observations suggest that prenatal exposure to X-ray irradiation damaged the development of vascular endothelial cells. A lack of endothelium development may further disrupt the organization of CNS cells and networks. In this vein, an improvement or enhancement of endothelial cell growth potentially improves CNS cell development and neural connections, which may be a possible therapeutic target to prevent development defects in the prenatal exposed subjects. On the other hand, it has been shown that a density of cortical neurons and astroglia was preserved in the postnatal rats following X-ray irradiation (1.0 Gy) despite a decrease in cortical thickness [45], [46]. These findings suggest that the local energy demand per unit volume was similar to that of non-exposed subjects. However, the total number of CNS cells decreased [45], and thus, the total energy level may be suppressed in exposed-animals. In this case, hypo-perfusion could result from a low metabolic demand in parenchyma tissue, which may be coupled to a total vascular length. Overall, these results strongly suggest that immature development of the brain vascular system and the related cerebral hypo-perfusion is of etiological importance in the delayed CNS development in subjects following prenatal exposure to X-ray irradiation. Future studies must address the molecular mechanism of CNS hypo-perfusion and its role in developing brains.

### Conclusion

In conclusion, the present study developed a novel analytical method to determine the mean RBC speed from a large number of traces from a population of RBC images based on the 2D FFT approach. The method is simple and robust and enables automated quantification with minimum computation time. Further, the present study showed the significant decline in microvascular perfusion in the cerebral cortex of prenatal-exposed rats. These findings are consistent with our previous report [2]. Thus, the present method is valuable to further our understanding of the causal relationship between the decline of cerebral microvascular perfusion and delayed CNS development in the subjects following prenatal exposure to X-ray irradiation.

## Materials and Methods

### Animal preparation

The study was carried out in accordance with the recommendations in the Guide for the Care and Humane Use of Laboratory Animals of the National Institutes of Health. All experimental protocols were approved by the Institutional Animal Welfare Committee (Permit Number: 09-1008-4). In accordance with a previous study [2], two pregnant female Sprague-Dawley rats (15th day of pregnancy, Japan SLC, Hamamatsu, Japan) were irradiated with a single whole-body X-ray at a dose of 1.5 Gy (200 kVp, 20 mA, 0.5 mm Cu plus 0.5 mm Al filter). At eight to nine weeks after birth, a total of four male rats (230 to 300 g) from each breed were used for the experiments. Nine age-matched male Sprague-Dawley rats (210 to 320 g, Japan SLC, Hamamatsu, Japan) were also used as the non-exposed control group for microvascular AVTT measurements in the cerebral cortex.

The animals were anesthetized with isoflurane, and endotracheal intubation was performed to allow for mechanical ventilation. Catheters were placed into the femoral vein and artery for drug administration and for arterial blood sampling and monitoring systemic arterial blood pressure, respectively. The animal head was then fixed with stereotaxic frame (SG-3N, Narishige, Tokyo, Japan) by holding the animal nose and ears. Three needles were placed into the both sides of underarm and back for electrocardiogram (ECG) recording, and rectal temperature was maintained at 37°C. The left side of the skull over the somatosensory cortex (3 mm by 5 mm) was thinned with a dental drill. After the surgery was completed, the inspired gas was switched to a mixture of air and O_2_ (30 to 35% total O_2_), and the end-tidal isoflurane concentration was adjusted to 1.4%. The respiratory parameter was adjusted based on the arterial blood gas conditions: pH = 7.554±0.041, PaCO_2_ = 32.3±1.0 mmHg, PaO_2_ = 161±7 mmHg, hematocrit = 38.5±2.5%, and glucose concentration  = 174±11 mg/dl in exposed group (N = 4), and pH = 7.471±0.024, PaCO_2_ = 34.6±2.8 mmHg, PaO_2_ = 136±23 mmHg, hematocrit  = 38.7±2.2%, and glucose concentration  = 208±31 mg/dl in non-exposed group (N = 9). There were significant differences in the conditions of blood pH, PaO_2_, and glucose concentrations between the exposed and non-exposed groups (p<0.05). Arterial blood pressure, measured with a blood pressure transducer (TSD104A, Biopac Systems, Inc., Goleta, CA), and heart rate, measured with an EEG amplifier (EEG100C, Biopac Systems, Inc.), were recorded throughout the experiments with a data acquisition system (MP150, Biopac Systems, Inc.) at a sampling rate of 100 Hz: mean arterial blood pressure (MABP)  = 106±11 and 99±7 mmHg, and heart rate  = 335±47 and 333±24 beats/min in exposed and non-exposed groups, respectively.

### Measurement of microvascular RBC speed

The cortical microvasculature was imaged through a thinned skull using a two-photon laser scanning fluorescent microscope (TCS SP5MP, Leica Microsystems GmbH, Wetzlar, Germany) at 900-nm excitation (Mai Tai HP, Spectra-Physics, Santa Clara, CA) with an emission band-pass filter of 655/50 nm. Qdot 655 (1 µM in saline, Invitrogen, San Diego, CA) was intravenously injected into the animal to fluorescently label blood plasma [30]. RBC speed was measured in microvessels that had a diameter of less than 6 µm (i.e., capillaries) and a straight section at least 50 µm long, located between a depth of 50 and 300 µm from the cortical surface. For each target vessel, line scanning was repeatedly performed along the length of the vessel at a rate of 1-4 lines/ms, and a total of 512 lines were used to make a single image (i.e., 512 by 512 pixels, see also Fig. 1A). The image consists of an x-axis as the time domain (0.25-1 ms/pixel, Δt) and y-axis as the spatial domain (0.06-0.20 µm/pixel, Δx). The field of view and line-scan average were manually adjusted (i.e., 2-8 scan averages were typically performed with a scan speed of 0.125 ms/line). By setting 512 pixels along the horizontal axis (Δt), the temporal window was accordingly 128-512 ms, which allowed accurate calculation of the RBC speed under our experimental conditions. However, the window size is not limited. Depending on the line-scan average, an even shorter time-window (e.g., 32 pixels) is also possible (Fig. S2).

Mean RBC speed (v) was determined with the following equations:
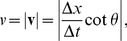
(1)where θ is the angle between the temporal frequency axis and a line perpendicular to the streaks (see Fig. 1B), and Δx and Δt are the spatial and temporal sampling intervals (i.e., a pixel width), respectively. Since Δx and Δt in Eq. (1) are given by the image acquisition parameters, θ is the only quantity that directly reflects the RBC speed. The angle θ was automatically calculated by detecting the angle having the maximum summation (g) of the power spectrum across all directions (from -0.5π to +0.5π) with a resolution of π/180 around a center of the frequency space (see Fig. 1C):
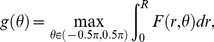
(2)where F(r, θ) is the transformation of a power spectrum image from Cartesian to polar coordinates. Two-dimensional linear interpolation was applied in the coordinate transformation. R was half of the pixel number, 255 in this study.

### Measurement of the AVTT

Time-lapse imaging of the cortical surface vasculature was performed at a rate of 7.1, 9.5, or 14.2 frame/sec using a single-photon excitation mode (488-nm Argon laser). The image was 512 by 512 pixels, and the in-plane resolution was 3.6 µm/pixel. Immediately after the initiation of time-lapse imaging (within 10 sec), a bolus injection of Qdot 655 (1 ml/kg body weight) was administered in the femoral vein. AVTT was determined by imaging plasma markers but not RBC. Because negligible differences in the AVTT were observed between the plasma and RBC markers (data not shown), the equivalent flow characteristics of the plasma and RBC were assumed in the experimental conditions in the comparison of the measured RBC speed using conventional microvascular perfusion measurements. A median filter was first applied to reduce the shot noise of the detector (photomultiplier). AVTT was then calculated by subtracting the appearance time of the fluorescent signals determined at venous segments by those of arterial segments. The appearance time is the earliest time point that both target pixel intensity and the subsequent five time-point intensities surpassed a threshold level (i.e., mean + 2 S.D. of pre-injection baseline intensity). The baseline intensity was calculated by averaging all intensities measured during the pre-injection periods on a single pixel basis. If there were no time-points that surpass the threshold, that pixel was excluded from further analysis. Finally, arterial and venous segments were manually extracted based on a spatial continuity of the measured appearance time along a longitudinal direction of the vessels, and the appearance time of each segment was reported by averaging all pixel data of the respective segments. Data were presented as mean±S.D. across the animals unless otherwise specified, and a Student's t-test was performed for statistical analysis to compare the radiation-exposed and non-radiation group data.

## Supporting Information

Figure S1 **Comparison of with and without cropping for RBC speed estimation.** (A) Raw image (512 by 512 pixels) captured by line scanning along a single vessel. The regions of interest in blue (cropped,128 by 128 pixels) and pink (non cropped, 512 by 128 pixels) were compared. (B) FFT. A single peak was consistently seen for both the cropped (blue) and non-cropped (pink) images. (C) Radon transform. Identical peak location was observed for the cropped (blue) and non-cropped (pink) images, but in the latter case the peak height was hidden by the components originating from non-vascular areas of the non-cropped image.
(PDF)

Figure S2 **Temporal window and estimated RBC speeds.** The dependence of RBC speed estimation on temporal window size was compared for the FFT and Radon transform methods. For less than 32 pixels (x-axis), both methods produced large variations away from the expected velocities (1 mm/s). Thus, a minimum of 32 pixels was needed to achieve accurate estimation. Note that the time window dependencies were similar for both the FFT and Radon transform methods.
(PDF)

Figure S3 **RBC speed histogram for the non-exposed animals (modified from** [**30**] **).** The RBC speed was directly measured by tracking the displacement of individual RBCs in the non-exposed rats [30] under similar experimental conditions to the present study. A mean speed of 1.5±0.4 mm/s was observed.
(PDF)
