# Supplementary figures and images for: Spatial Frequency-Based Analysis of Mean Red Blood Cell Speed in Single Microvessels: Investigation of Microvascular Perfusion in Rat Cerebral Cortex
Source: PLoS One. 2011 Aug 24;6(8):e24056. doi: 10.1371/journal.pone.0024056 (PMC3161111; doi:10.1371/journal.pone.0024056)

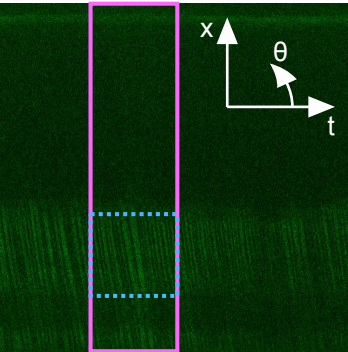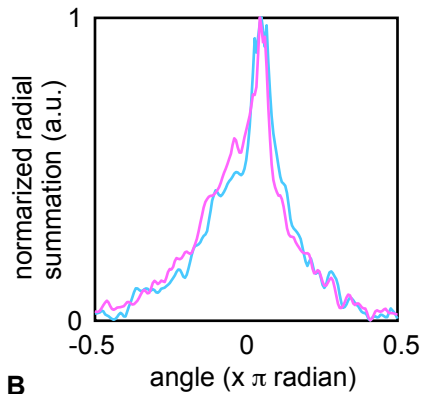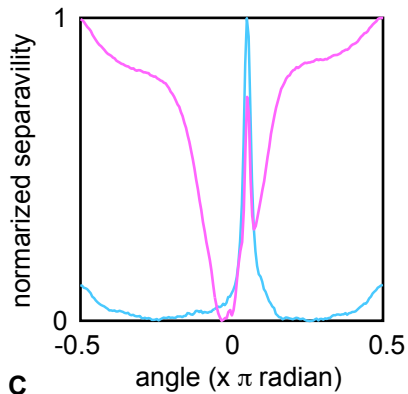

Supplement: Figure S1 — Comparison of with and without cropping for RBC speed estimation. (A) Raw image (512 by 512 pixels) captured by line scanning along a single vessel. The regions of interest in blue (cropped,128 by 128 pixels) and pink (non cropped, 512 by 128 pixels) were compared. (B) FFT. A single peak was consistently seen for both the cropped (blue) and non-cropped (pink) images. (C) Radon transform. Identical peak location was observed for the cropped (blue) and non-cropped (pink) images, but in the latter case the peak height was hidden by the components originating from non-vascular areas of the non-cropped image. (PDF) [file pone.0024056.s001.pdf]

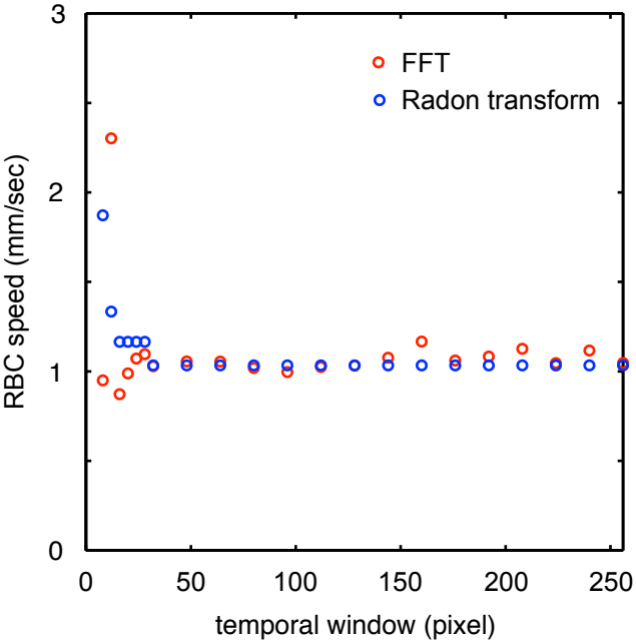

Supplement: Figure S2 — Temporal window and estimated RBC speeds. The dependence of RBC speed estimation on temporal window size was compared for the FFT and Radon transform methods. For less than 32 pixels (x-axis), both methods produced large variations away from the expected velocities (1 mm/s). Thus, a minimum of 32 pixels was needed to achieve accurate estimation. Note that the time window dependencies were similar for both the FFT and Radon transform methods. (PDF) [file pone.0024056.s002.pdf]

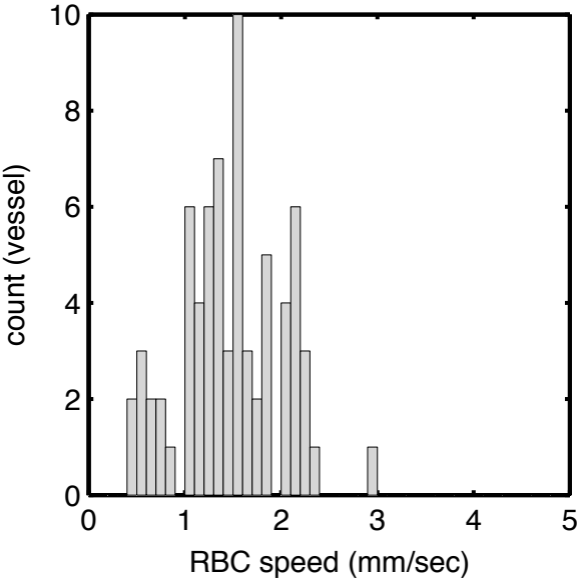

Supplement: Figure S3 — RBC speed histogram for the non-exposed animals (modified from [30] ). The RBC speed was directly measured by tracking the displacement of individual RBCs in the non-exposed rats [30] under similar experimental conditions to the present study. A mean speed of 1.5±0.4 mm/s was observed. (PDF) [file pone.0024056.s003.pdf]
